# Effects of Zinc Oxide Nanoparticle Exposure on Human Glial Cells and Zebrafish Embryos

**DOI:** 10.3390/ijms241512297

**Published:** 2023-08-01

**Authors:** Vanessa Valdiglesias, Anabel Alba-González, Natalia Fernández-Bertólez, Assia Touzani, Lucía Ramos-Pan, Ana Teresa Reis, Jorge Moreda-Piñeiro, Julián Yáñez, Blanca Laffon, Mónica Folgueira

**Affiliations:** 1 Universidade da Coruña, Grupo NanoToxGen, Centro Interdisciplinar de Química e Bioloxía—CICA, Departamento de Biología, Facultad de Ciencias, Campus A Zapateira s/n, 15071 A Coruña, Spain; vvaldiglesias@udc.es (V.V.); natalia.fernandezb@udc.es (N.F.-B.); assia.touzani@udc.es (A.T.); lucia.ramosp@udc.es (L.R.-P.); 2 Instituto de Investigación Biomédica de A Coruña (INIBIC), Oza, 15071 A Coruña, Spain; 3 Universidade da Coruña, Grupo NEUROVER, Centro Interdisciplinar de Química e Bioloxía—CICA, Rúa As Carballeiras, 15071 A Coruña, Spain; anabel.albag@udc.es (A.A.-G.); julian.yanez@udc.es (J.Y.); m.folgueira@udc.es (M.F.); 4 Universidade da Coruña, Grupo NEUROVER, Facultad de Ciencias, Campus A Zapateira s/n, 15071 A Coruña, Spain; 5 EPIUnit—Instituto de Saúde Pública, Universidade do Porto, Rua das Taipas 135, 4050-600 Porto, Portugal; ana.reis@insa.min-saude.pt; 6 Laboratório para a Investigação Integrativa e Translacional em Saúde Populacional (ITR), Rua das Taipas 135, 4050-600 Porto, Portugal; 7 Environmental Health Department, National Institute of Health, Rua Alexandre Herculano, 321, 4000-055 Porto, Portugal; 8 Universidade da Coruña, Grupo Química Analítica Aplicada (QANAP), Instituto Universitario Medio Ambiente (IUMA), Departamento de Química, Facultad de Ciencias, Campus A Zapateira s/n, 15071 A Coruña, Spain; jorge.moreda@udc.es; 9 Universidade da Coruña, Grupo DICOMOSA, Centro Interdisciplinar de Química e Bioloxía—CICA, Departamento de Psicología, Facultad de Ciencias de la Educación, Campus Elviña s/n, 15071 A Coruña, Spain

**Keywords:** cytotoxicity, human A172 glial cells, zebrafish, zinc oxide nanoparticles, Zn^2+^ ions

## Abstract

Zinc oxide nanoparticles (ZnO NPs) are among the most widely used nanomaterials. They have multiple applications in cosmetics, textiles, paints, electronics and, recently, also in biomedicine. This extensive use of ZnO NPs notably increases the probability that both humans and wildlife are subjected to undesirable effects. Despite being among the most studied NPs from a toxicological point of view, much remains unknown about their ecotoxicological effects or how they may affect specific cell types, such as cells of the central nervous system. The main objective of this work was to investigate the effects of ZnO NPs on human glial cells and zebrafish embryo development and to explore the role of the released Zn^2+^ ions in these effects. The effects on cell viability on human A172 glial cells were assessed with an MTT assay and morphological analysis. The potential acute and developmental toxicity was assessed employing zebrafish (*Danio rerio*) embryos. To determine the role of Zn^2+^ ions in the in vitro and in vivo observed effects, we measured their release from ZnO NPs with flame atomic absorption spectrometry. Then, cells and zebrafish embryos were treated with a water-soluble salt (zinc sulfate) at concentrations that equal the number of Zn^2+^ ions released by the tested concentrations of ZnO NPs. Exposure to ZnO NPs induced morphological alterations and a significant decrease in cell viability depending on the concentration and duration of treatment, even after removing the overestimation due to NP interference. Although there were no signs of acute toxicity in zebrafish embryos, a decrease in hatching was detected after exposure to the highest ZnO NP concentrations tested. The ability of ZnO NPs to release Zn^2+^ ions into the medium in a concentration-dependent manner was confirmed. Zn^2+^ ions did not seem entirely responsible for the effects observed in the glial cells, but they were likely responsible for the decrease in zebrafish hatching rate. The results obtained in this work contribute to the knowledge of the toxicological potential of ZnO NPs.

## 1. Introduction

Zinc oxide (ZnO) nanoparticles (NPs) are among the most broadly used metal oxide NPs in the world market. Specifically, these NPs are used in a wide range of everyday products, such as cosmetics, toothpaste, sunscreens, fillers in medical materials, ornamental plants, textiles, paints, and electronics and many more. They are also used in the food industry, for instance as an antibacterial compound in food packaging or, in agriculture, as an alternative to conventional zinc fertilizers to increase the availability of Zn for plants [1,2,3,4,5]. Recently, ZnO NPs have been demonstrated to have antibacterial properties when conjugated with other compounds, so they are being investigated as nanocomposites for clinical and environmental use [6,7,8,9]. The average industrial production of ZnO NPs is estimated at around 550–33,400 annual tonnes [10]. This extensive use of ZnO NPs notably increases the probability that both humans and wildlife are subjected to undesirable effects.

The toxicological research on ZnO NPs is far behind the extent of their application, on account of the conventional view that zinc oxide is non-toxic [11]. The influence of ZnO NPs on the human body is still not clear [12,13]. Due to their small size, these NPs can cross biological barriers, such as the blood–brain barrier, and reach the central nervous system, as demonstrated in experimental animals after oral [14] and inhalatory [15] administration. They may also cause biochemical alterations in neurological and immunological condition and the male reproductive organs, mainly related to oxidative stress and inflammation, demonstrated in rats after oral exposure [16]. Still, little is known about the possible effects or action mechanism of ZnO NPs on nervous system cells. From an ecotoxicological point of view, ZnO NPs present in the environment might pose risks to different ecosystems. Environmental levels of these NPs were reported to be in the range of 3.1–31 μg/kg soil and 76–760 μg/L wastewater [17,18], causing a considerable chemical and biological impact on these systems (reviewed in [19]). This is one of the main reasons why these metal oxide NPs have attracted much attention in recent decades. 

A number of studies reported harmful cellular effects of ZnO NPs in different cell systems, including genetic damage, alterations in the function and structure of cellular organelles, cell cycle disruptions, induction of cell death, cellular membrane impairment, and reactive oxygen species (ROS) production [3,13,20,21,22]. However, most of these studies did not discard the potential interference of the ZnO NPs with the reagents or detection systems used in the common toxicological tests, which has been demonstrated to be highly decisive in the observed results [23,24]. This interference may lead to false negative or positive results. Moreover, inflammation and oxidative stress are among the main action mechanisms often suggested to be behind the toxic effects of ZnO NPs reviewed in [25]. However, very few studies have addressed the role of the Zn^2+^ ions released from the ZnO NP surface on these toxic effects, particularly on the nervous system and during development. Thus, previously reported results of ZnO NP toxicity might be due to either the ZnO NPs themselves, the dissolved ions, or both. In this context, Keerthana and Kumar [25] recently concluded, after reviewing 277 studies, that ZnO NPs could be beneficial in the treatment of various diseases, but their safety at effective concentrations should be thoroughly evaluated. 

Glial A172 cells, an astrocytoma non-tumorigenic cell line, are well recognized as a human glial model for neurotoxicity testing [26,27]. They are a type of brain cells involved in metabolic, homeostatic, and immunological functions. As a result, astrocyte dysfunction or physiological reaction to an injury can amplify neuronal damage [4].

Zebrafish (*Danio rerio*, Cypriniform) is an appealing in vivo model to assess the hazards of both conventional chemicals and nanomaterials in ecotoxicology [28,29,30,31]. It is considered an excellent animal model due to its gene similarities with humans, small size, very high reproducibility, rapid development, and the transparency of embryos, amongst other characteristics [32,33]. In recent years, toxicity tests using zebrafish embryos have become popular, as they are cost-efficient, allowing highly reproducible results to be obtained quite quickly, and raise fewer ethical concerns than tests involving older stages or other species, such as mammals [33,34,35]. Because of their fast development and transparency, in addition to lethal toxicity, zebrafish embryos allow assessment of sublethal effects by analyzing the occurrence of malformations [33,36]. So, all in all, zebrafish is a powerful model that facilitates important advances in the daunting task of testing chemical and drug toxicity [32].

The main objective of this work was to investigate the effects of ZnO NPs on glial cells and zebrafish embryo development and to determine the role of released Zn^2+^ ions in these effects by employing the water-soluble salt zinc sulfate [37]. For this, the release of Zn^2+^ ions from ZnO NPs in water solutions was first measured with flame atomic absorption spectrometry (FAAS). Cells and zebrafish embryos were treated with ZnO NPs and zinc sulfate, the latter at concentrations that equaled the number of Zn^2+^ ions released by the tested concentrations of ZnO NPs. Cellular effects were evaluated in glial A172 cells by morphological analysis and the MTT assay. Acute toxicity and teratogenicity were tested in zebrafish by recording viability and hatching rate during the first 96 h of development and analyzing the presence of morphological malformations.

## 2. Results

### 2.1. Characterization of ZnO NPs

The ZnO NPs employed in this study are less than 100 nm spherical NPs with a surface area of 10–25 m^2^/g (data provided by the manufacturer). The results obtained from the analysis of hydrodynamic size and zeta potential are shown in Table 1. To confirm the stability of the NP dispersion throughout the in vitro and in vivo experiments, both parameters were calculated at the highest NP concentration from 0 up to 48 h in cell culture medium and up to 96 h in dechlorinated water. The dispersion of ZnO NPs was quite stable, with hydrodynamic sizes ranging between 273.97 nm and 315.01 nm in cell culture medium and between 238.15 and 301.36 nm in dechlorinated water. Zeta potential was also stable in dechlorinated water, always with negative charge and values between −23.23 and −18.22 mV. However, it was more variable in cell culture medium, with low absolute values at short times and higher negative values at longer times.

### 2.2. Zn^2+^ Ion Release

**Figure 1:**
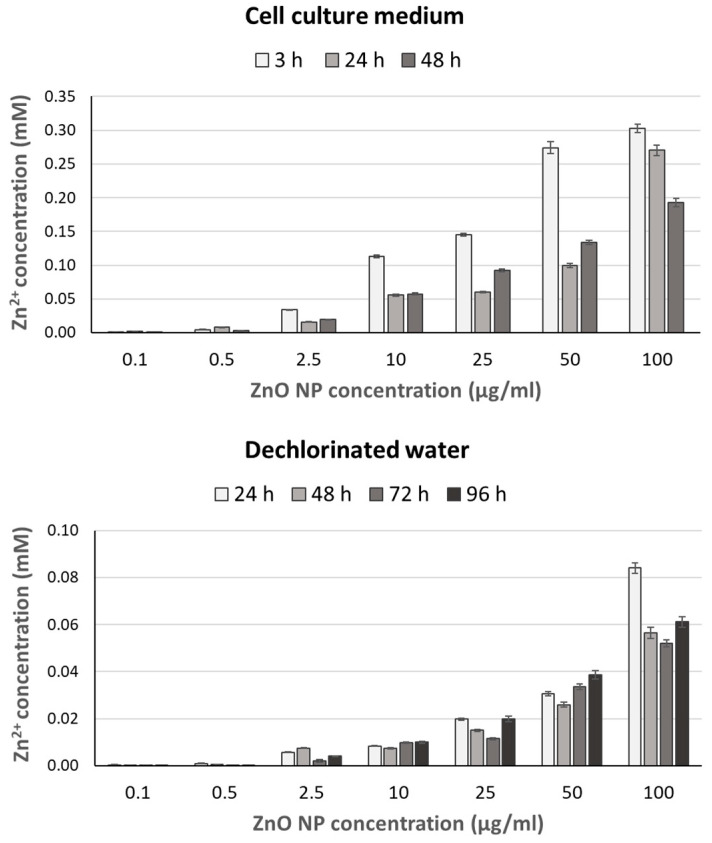
Analysis of Zn^2+^ ions released from ZnO NPs in cell culture medium and dechlorinated water. Bars represent the standard deviation.

The release of zinc ions from the ZnO NPs was quantified in cell culture medium and dechlorinated water after different incubation times according to FAAS. Results obtained from these experiments are shown in Figure 1. Notable concentrations of dissolved Zn^2+^ ions were observed both when ZnO NPs (from 2.5 µg/mL onwards) were suspended in medium (for 3, 24, and 48 h) and in water (for 24, 48, 72, and 96 h), increasing with NP dose in both cases. Concentrations observed were always higher for the shortest treatment than for the longer ones, particularly marked in cell culture medium.

### 2.3. Cellular Morphology

**Figure 2:**
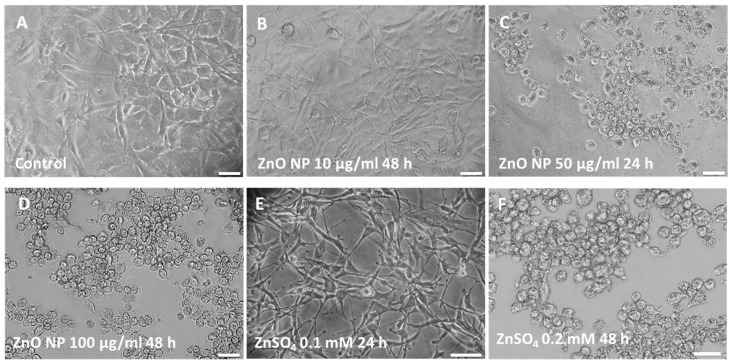
Morphology of A172 glial cells subjected to different treatments: control (**A**), treated with ZnO NPs at 10 µg/mL (**B**), 50 µg/mL (**C**), and 100 µg/mL (**D**) and treated with ZnSO_4_ at 0.1 mM (**E**) and 0.2 mM (**F**). Scale bar: 50 µm.

Figure 2 shows the comparative morphologies of unexposed and ZnO NP-exposed (0.1–100 µg/mL) A172 glial cells. Morphological alterations were observed after 24 and 48 h treatments with concentrations from 50 µg/mL ZnO NPs and above. Specifically, cells retracted to a spherical shape and detached from the substratum, forming clusters.

To investigate the potential role of the Zn^2+^ ions released from the ZnO NPs in the effects we observed, A172 cells were treated with zinc sulfate (ZnSO_4_) to obtain Zn^2+^ ions at concentrations comparable to those existing in the ZnO NP suspensions, according to FAAS results, i.e., 0.005 to 0.5 mM. Cells exposed to ZnSO_4_ showed morphological alterations only at the highest concentrations (from 0.2 mM and above) after 24 and 48 h of exposure (Figure 2).

### 2.4. Cell Viability Assay

**Figure 3:**
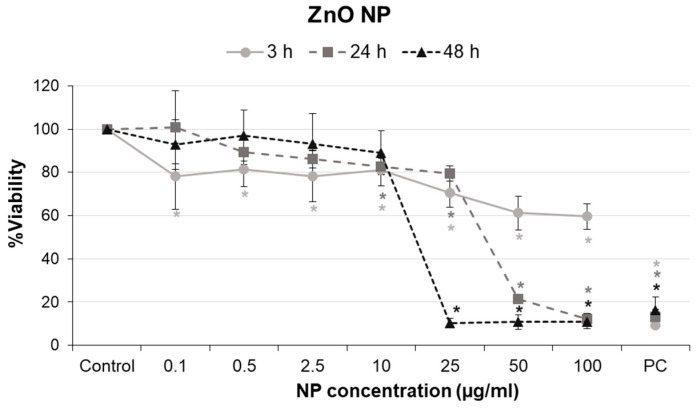
Cytotoxicity of ZnO NPs in A172 glial cells at different exposure times. PC: positive control. Bars represent mean ± standard error. * *p* < 0.05, significant difference regarding the corresponding control.

Once the interference of ZnO NPs with the MTT methodology was removed by introducing additional steps in the traditional protocol (supplementary material, Figure S2), the modified MTT protocol was used to evaluate the effects of ZnO NPs on the viability of glial A172 cells (Figure 3). After 3 h treatments, ZnO NPs induced a slight dose-dependent decrease in viability of glial cells (r = −0.706, *p* < 0.01). Statistically significant differences with respect to the control were achieved at all concentrations tested, although viability levels only decreased below 70% at the two highest concentrations assayed.

After 24 h of exposure, cell viability values remained above 80% up to 25 µg/mL. At higher concentrations, they dramatically dropped to values close to 20% and 10% at 50 and 100 µg/mL, respectively. A significant dose–response relationship was also obtained in this case (r = −0.832; *p* < 0.01). A similar behavior was observed after 48 h treatments (r = −0.766; *p* < 0.01), with no effect on cell viability at low concentrations but a marked decrease from 25 µg/mL and above, decreasing to 10% viability at higher doses.

From the data obtained in the MTT assays, IC_50_ values were calculated for each treatment time, obtaining the results summarized in Table 2. IC_50_ decreased progressively with the increase in treatment time.

**Figure 4:**
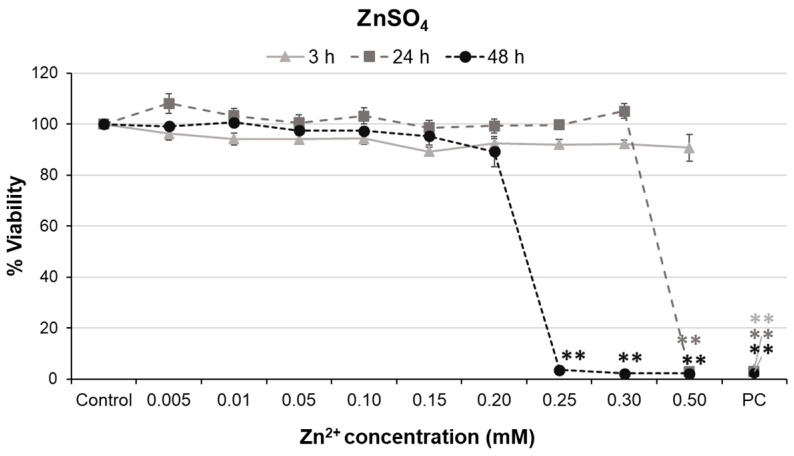
Cytotoxicity of ZnSO_4_ in A172 glial cells at different exposure times. PC: positive control. Bars represent mean ± standard error. ** *p* < 0.01, significant difference regarding the corresponding control.

A172 cells were also treated with ZnSO_4_ at concentrations ranging from 0.005 to 0.5 mM. Although significant concentration–response relationships were observed at the three times tested (r = −0.326, *p* < 0.01 for 3 h; r = −0.463, *p* < 0.01 for 24 h; r = −0.746, *p* < 0.01 for 48 h), significant decreases in cell viability were only found at the highest concentration tested after the 24 h treatment (0.5 mM), and from 0.25 mM and above after the 48 h treatment (Figure 4). No significant cytotoxicity was observed at 3 h. The calculated IC_50_ value for the 3 h treatment was notably high and decreased progressively at 24 and 48 h (Table 2). The IC_50_ concentrations for ZnSO_4_ were much higher and contained more dissolved Zn^2+^ than those for ZnO NPs (at the corresponding treatment times), according to the results presented in Figure 1. These results confirm that Zn^2+^ ions cannot be entirely responsible for the decrease in cell viability observed after ZnO NP treatments.

### 2.5. Fish Embryo Acute Toxicity Assay

**Figure 5:**
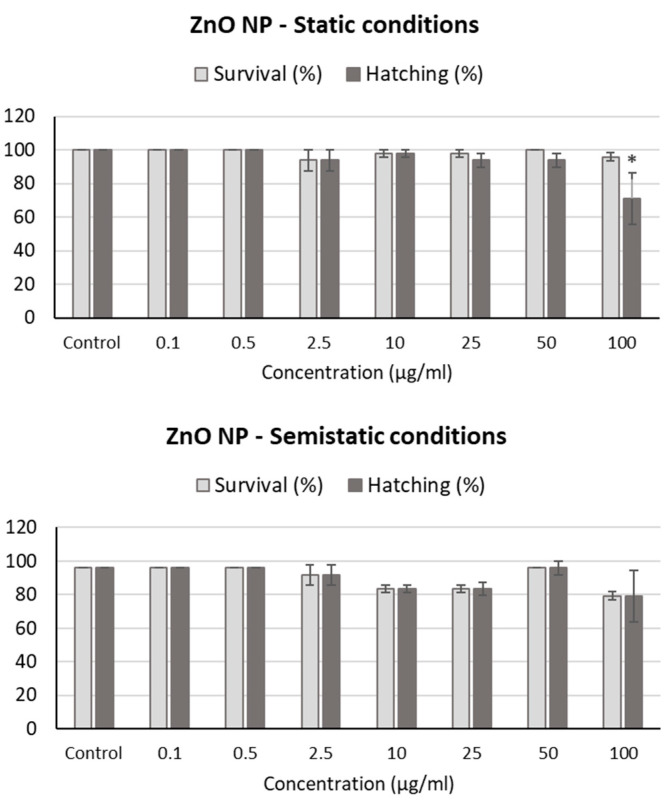
Survival and hatching in zebrafish embryos exposed to ZnO NPs for 96 h. * *p* < 0.05; significant difference regarding the corresponding control.

To test ZnO NP acute toxicity in zebrafish embryos, static experiments, in which the ZnO NP suspensions were not changed throughout the experiments, were initially performed. Since ZnO NPs tended to settle on the bottom of the wells after 24 h of exposure at high concentrations, semistatic experiments with daily medium replacement were additionally performed, although they did not show significant differences when compared to the static ones (Figure 5). ZnO NPs exhibited no significant mortality compared to controls at any of the concentrations tested, considering mortality as the presence of any of the five endpoints analyzed (i.e., coagulated fertilized eggs, lack of heartbeat, presence of edema, absence of somites, and non-detachment of the tail). Even though there was a slightly higher mortality at the highest concentrations of ZnO NPs, especially in the semistatic experiments, there was no statistical significance in the results. Additionally, a decrease in the hatching rate for the embryos exposed to ZnO NPs was observed, especially for the highest concentration tested, in both the static and semistatic experiments, but statistical significance was only reached in the former.

**Figure 6:**
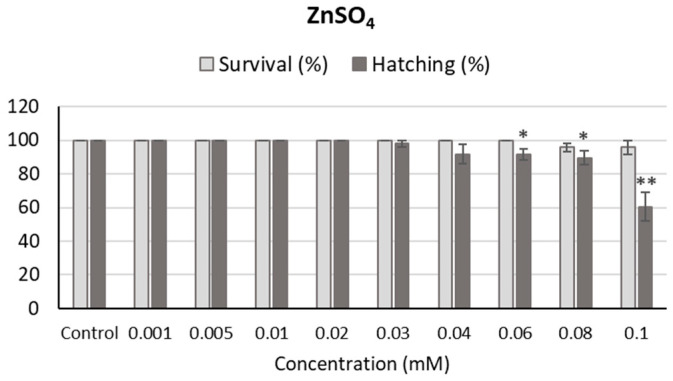
Survival and hatching in zebrafish embryos exposed to ZnSO_4_ for 96 h. * *p* < 0.05; ** *p* < 0.01, significant difference regarding the corresponding control.

Zebrafish embryos were also exposed to ZnSO_4_; the concentrations tested ranged from 0.001 to 0.1 mM, selected according to FAAS results in dechlorinated water (Figure 6). Given that ZnSO_4_ does not precipitate under the conditions tested and that semistatic experiments are potentially more invasive, only static experiments were performed for ZnSO_4_. Survival was higher than 95% at 96 h post-fertilization (hpf) for all concentrations tested, whereas hatching decreased in a dose-dependent manner (r = −0.770, *p* < 0.05), reaching statistical difference with regard to the control at concentrations higher than 0.04 mM. Thus, these results show that, although ZnSO_4_ does not produce a decrease in survival, it does compromise hatching at high concentrations.

### 2.6. Zebrafish Morphological Alterations

**Figure 7:**
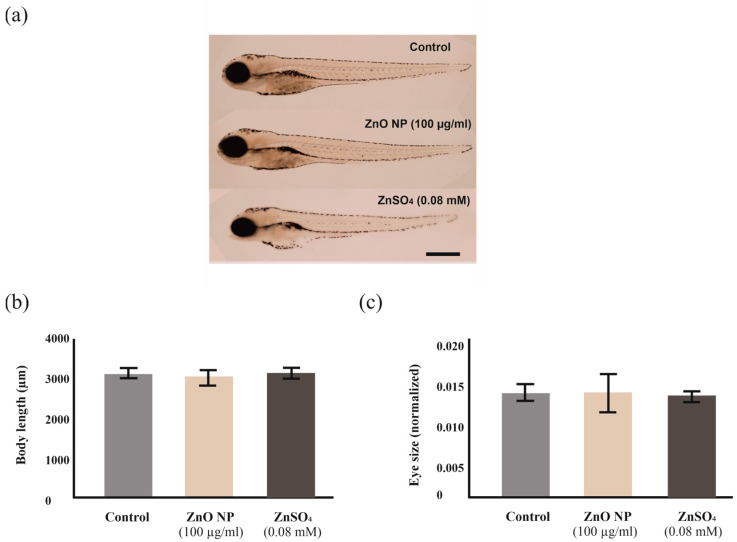
Analysis of zebrafish embryos morphology after exposure to ZnO NPs and ZnSO_4_. (**a**) Images showing the general morphology of zebrafish embryos after 96 h exposure to dechlorinated water only (control; top), ZnO NPs (100 µg/mL, middle) and ZnSO_4_ (0.08 mM; bottom). Selected individuals are representative of each group. Scale bar 500 µm. (**b**) Body length and (**c**) normalized eye size. Bars represent mean ± standard error.

In order to detect the presence of sublethal toxicity, various morphological characteristics of the embryos exposed to ZnO NPs (100 µg/mL) and ZnSO_4_ (0.08 mM, corresponding to the maximum concentration of Zn^2+^ released by the ZnO NPs in dechlorinated water) were analyzed at 96 hpf, including body length and eye size (normalized to body length). Exposure to ZnO NPs or ZnSO_4_ did not induce alterations in the morphological features assessed (body length and normalized eye size) compared to control embryos (Figure 7a–c). Still, it was noticed that a small percentage of embryos treated with ZnSO_4_ (20%) showed alterations in the dorsal craniofacial region (not shown), which were not observed in those exposed to ZnO NPs or in controls. Other craniofacial features of embryos exposed to ZnSO_4_, such as the morphology of the lower jaw, were normal (Figure 7a).

## 3. Discussion

Despite being “Generally Recognized as Safe” (GRAS) by the United States Food and Drug Administration (FDA) [38], the use of ZnO NPs has been increasingly associated with reports of toxicity and side effects in different organisms and ecosystems. Much remains to be explored regarding their action mechanisms and potential harmful effects, particularly on the nervous system and during development. Therefore, in this work, we evaluated the cellular and developmental effects of ZnO NP exposure in vitro on human glioblastoma A172 cells and in vivo on zebrafish embryos. It has been previously suggested that Zn^2+^ ions mediate in ZnO NP toxicity [39,40], so their role in the effects we observed after ZnO NP exposure was also addressed by using their ionic counterpart, the water-soluble salt ZnSO_4_. The ZnSO_4_ concentration range was chosen according to FAAS results. We observed that the number of Zn^2+^ ions released by the ZnO NPs into the medium increased in a dose-dependent manner, being markedly higher at 3 h than at 24 or 48 h. This peak after 3 h may occur due to the ions binding to macromolecules present in the medium a short time after being released, and these macromolecules were subsequently removed from the solution by high-speed centrifugation (22,000× *g*) prior to conducting FAAS. The release of Zn^2+^ ions from ZnO NPs is well documented in the literature [41,42,43,44,45].

Prior to evaluating the effects of ZnO NPs on glial cells and zebrafish embryos, their physicochemical properties were characterized as they may significantly influence their biological behavior and toxicological profile [46,47]. Results showed that the hydrodynamic size of the ZnO NPs used in this work remained stable and did not agglomerate in either the cell culture medium or the dechlorinated water, with minimal variations over time (up to 96 h). Also, zeta potential remained quite stable in dechlorinated water but was more variable in cell culture medium. This parameter presented higher negative values at longer exposure times, indicating that ZnO NPs might experience slight modifications in their surface reactivity with time in contact with the components of the culture medium. This would explain the high stability of the dispersion in the long term, involving a lower tendency to form aggregates of ZnO NPs due to the increase in electrostatic repulsion between particles [46,48,49].

Modifications of A172 glial cell morphology as a result of ZnO NPs or zinc sulfate exposure were analyzed in this study. Our results showed that, under specific conditions (24 and 48 h treatments from 50 µg/mL ZnO NPs, and 48 h from 0.2 mM ZnSO_4_ onwards), cells growing as a monolayer detached from the substrate, rounded up, and exhibited morphological alterations. Our results are in line with a previous study in which different degrees of deformation, including rounding up and floating, full nuclear condensation, and formation of several intracellular vacuoles, were observed after treating rat PC12 neuronal cells with ZnO NPs (8 and 16 µg/mL) for 6 h [50]. These changes in cell morphology may be caused by reorganization of the cytoskeleton, which plays key roles in the maintenance of cell shape and adhesion, together with other molecules. As various types of cell death are preceded by a reorganization of the cytoskeleton, whether the observed morphological changes occur because cells exit the cell cycle and enter an apoptotic pathway remains to be analyzed.

In recent years, one of the main concerns in the nanotoxicology field is the suitability of standard in vitro toxicity methods for nanotoxicity screening. It is questionable whether they are adequate to evaluate NP effects, due to the possible interference of the NPs with different assay components or detection methods, which may result in a lack of reliability of the results obtained [51,52,53,54]. Potential interference of metal NPs with the traditional protocol of the MTT assay has also been described [55], which included interference with light absorption, catalytic interference through chemical reactions between NPs and reagents, and/or dye adsorption on the NP surface [56,57,58]. In the present work, both light absorption and catalytic interference were confirmed, so they were corrected with the addition of supplementary washes and centrifugation steps (Supplementary Material Figure S2). Our findings show that it is critical to test the interference of NPs prior to carrying out toxicity assessments in order to avoid false positive or false negative results. 

Results obtained from the viability analysis showed a dose- and time-dependent effect of ZnO NP exposure on glial cells. We observed mild cytotoxicity (around 60% of viability) in the short-term (3 h) at the highest concentrations and high cytotoxicity (up to 90% of mortality) in the medium- (24 h) and long-term (48 h) from concentrations 50 and 25 µg/mL and above, respectively. According to ISO 10993-5 (International Organization for Standardization, 2009) [59], a reduction in cell viability greater than 30% is considered a cytotoxic effect. Accordingly, ZnO NPs would be considered cytotoxic to A172 astrocytes at high concentrations (>10 μg/mL) in a time- and concentration-dependent manner. In addition, IC_50_ showed a strong time dependence. The IC_50_ values were notably higher after applying the modified protocol than those calculated from the standard protocol (68.02 ± 10.45 µg/mL at 3 h, 21.53 ± 1.12 µg/mL at 24 h, and 21.13 ± 1.24 µg/mL at 48 h). This confirms that results based on the standard protocol overestimate the damage induced (false positive results, particularly at 3 and 24 h) and supports once more the importance of ruling out NP interference for correct and reliable testing.

Although several authors described a dose-dependent decrease in cell viability for various cell types exposed to ZnO NPs (reviewed in [3,21]), our study is among the very few that addressed the ZnO NP toxicity in human nervous cells. In this regard, Leung et al. determined that exposure of human astrocytoma U87 cells for 48 h to the same ZnO NP (concentrations > 1 μg/mL) induced a concentration-related decrease in cell survival (MTT assay), reporting an IC_50_ value similar to the one obtained in this study [60]. Another recent study demonstrated a similar effect on viability (MTT assay) in a primary culture of rat astrocytes, showing a decrease in cell viability dependent on the dose and exposure time, with cytotoxic effects from 30 µg/mL onwards after 24 h of ZnO NP exposure [61]. However, the IC_50_ value obtained after 24 h exposure in the study (60 μg/mL) was almost twice the one we observed in the present study (34.76 μg/mL), indicating that rat astrocytes are less sensitive to ZnO NP toxicity than human glial A172 cells. Sharma et al. also found a dose-dependent increase in cell death for mouse N9 microglia after the 24 h treatment with ZnO NPs (>50 nm, 1–100 μg/mL) [62]. However, in this case, a more extreme cytotoxic effect was observed than in astrocytes, reflected in the lower IC_50_ value (6.612 μg/mL ZnO NP). This difference in cytotoxicity for N9 microglia and A172 astrocytes may be due to different sensitivity for both cell types but also to the low stability and higher tendency to agglomerate of these NPs in N9 culture medium, as indicated by their hydrodynamic size and zeta potential (584 nm and −15.8 mV, respectively). Still, it should be noted that none of the mentioned studies tested the possible interference of ZnO NPs with the cytotoxicity tests used. Based on the results we obtained, it is highly likely that, at least in those studies using the MTT colorimetric assay [41,60,61], the results might not be entirely reliable due to the possible interference of the ZnO NPs with the methodology.

When glial cells were treated with Zn^2+^ ions (0.005–100 mM of zinc sulfate), a significant decrease in cell viability was observed just at Zn^2+^ concentrations greater than 0.3 and 0.2 mM for 24 and 48 h, respectively. These concentrations of released Zn^2+^ were only reached at the highest concentration of ZnO NPs (100 µg/mL) for the longest exposure times. We observed cytotoxicity at low concentrations of ZnO NPs, even when they had much less released Zn^2+^ present in the medium than those concentrations of zinc sulfate that are cytotoxic. These results indicate that the release of Zn^2+^ ions from the ZnO NPs does not explain, at least not completely, their effects on glial cell viability. Indeed, other modes of action independent of the presence of dissolved ions that could not be discarded in the present work, including ROS production and induction of oxidative stress, have been previously suggested for ZnO NPs [41,63,64,65].

Since the zebrafish embryo is a good model to assess the toxicity of NPs [66], it was used in this study to analyze lethal and sublethal toxic effects induced by ZnO NPs during development. According to the results obtained from the acute toxicity assay, exposure to ZnO NPs did not reduce survival in zebrafish embryos. Previous studies reported similar results [67,68,69]. However, significant effects on zebrafish embryos’ survival have been shown for other ZnO NPs, in these cases suggested likely to be caused by an increase in oxidative stress (reviewed in [70]). Multiple parameters influence the toxicity of ZnO NPs, such as size, coating, charge, and surface charge [70,71,72,73]. Thus, differences described in their toxicity are likely to reflect the variability in the characteristics of the tested nanomaterials, as well as, to some extent, the variability in experimental conditions [70]. 

A very commonly reported sublethal effect for ZnO NPs in zebrafish embryos is a decrease in the hatching rate [71,74,75]. Zebrafish hatching occurs as a result of the digestion of the chorion (egg envelope) by hatching enzymes and embryo movement [76]. The delay in hatching induced by ZnO NPs is suggested to be caused, at least partially, by the disturbance of the hatching enzymes, which are Zn-metalloproteases secreted by the embryo’s hatching gland [77]. We observed a slight decrease in the hatching rate for concentrations of ZnO NPs higher than 2.5 µg/mL, being statistically significant for 100 µg/mL (static experiments). The same trend was obtained for the ZnSO_4_ treatments, statistically significant from 0.06 mM and above. This Zn^2+^ concentration was released from the ZnO NPs at 100 µg/mL. Therefore, Zn^2+^ ions may be responsible for the decrease in the hatching rate caused by ZnO NP exposure.

Exposure to ZnO NPs did not affect the morphological parameters analyzed in the present study, as also observed for other ZnO NPs [68,78]. Other studies, however, found that exposure to ZnO NPs induces important morphological alterations reviewed in [70], including edema, shorter body length, and altered body curvature. We scored edema as one of the endpoints in the acute toxicity study, considering that an embryo showing this feature would be counted as dead [79]. In any case, we did not observe a higher prevalence of edema in ZnO NP-exposed embryos compared to controls. Nevertheless, further studies would be necessary to assess whether ZnO NPs can induce more subtle toxic effects than those pinpointed by analyzing general morphology.

## 4. Materials and Methods

### 4.1. Chemicals

ZnO NPs (CAS No. 1314-13-2), 3-(4,5-dimethylthiazol-2-yl)-2,5-diphenyl tetrazolium bromide (MTT) (CAS No. 298-93-1), dimethyl sulfoxide (DMSO) ACS reagent ≥ 99.9% (CAS No. 67-68-5), and phosphate-buffered saline (PBS) were purchased from Sigma-Aldrich Co (Merck KGaA, Darmstadt, Alemania). Triton X-100 (CAS no. 9002-93-1) was purchased from PanReac AppliChem (Castellar del Vallès, Spain), and zinc sulfate heptahydrate (ZnSO_4_·7H_2_O, CAS No. 7446-20-0, hereafter ZnSO_4_) was obtained from Scharlab S.L. (Barcelona, España). 3,4-Dichloroaniline solution (CAS No. 95-76-1) was purchased from Thermo Fisher Scientific Inc. (Uppsala, Sweden).

### 4.2. Nanoparticle Suspension: Preparation and Characterization

A stock suspension of ZnO NPs (final concentration 100 µg/mL) was prepared in either dechlorinated sterile water or in complete A172 cell culture medium (see composition in Section 4.3). Before each treatment, this suspension was ultrasonicated on ice with a 2.5 mm probe (Sonoplus mini 20, Bandelin, Berlin, Germany) at 30 W for 5 min (0.5 min on and 1 min off twice, plus 2 min on) and diluted to prepare the different NP concentrations tested. Average hydrodynamic size and size distribution and zeta potential of particles in both dechlorinated water and cell culture medium were determined by dynamic light scattering (DLS) and mixed-mode measurement phase analysis light scattering (M3-PALS), respectively, using a Zetasizer Nano-ZS (model ZEN 3600, Malvern Instruments Ltd., Worcestershire, UK) equipped with 4.0 mW, 633 nm laser. These analyses were carried out at different exposure times, specifically up to 72 h for cell culture medium and up to 96 h for dechlorinated water, to determine the temporal evolution of the NP stability and their state of aggregation during the subsequent in vitro and in vivo experiments. 

### 4.3. Cell Culture and Treatments

The human glioblastoma A172 cell line (ECACC 88062428) was obtained from the European Collection of Cell Cultures and cultured in DMEM medium with 1% L-glutamine and 1% antibiotic and antimycotic solution and supplemented with 10% heat-inactivated fetal bovine serum (FBS). Cells were incubated in a humidified atmosphere with 5% CO_2_ at 37 °C. Prior to the experiments, 2 × 10^4^ cells per well were seeded in 96-well plates (flat bottom) and allowed to adhere for 24 h at 37 °C. For the morphological analysis and MTT assay, these cells were incubated at 37 °C for 3, 24, or 48 h in the presence of 0.1, 0.5, 2.5, 10, 50, and 100 µg/mL ZnO NP concentrations (i.e., 0.03–31.25 µg/cm^2^), or the control solutions. Complete medium was used as a negative control in all experiments. Triton X-100 (1%) was used as a positive control in the MTT viability assay.

### 4.4. Morphological Analysis

Changes in cell morphology in A172 glial cells induced by ZnO NPs were observed under a light microscope (Nikon TMS, Nikon Corporation, Tokyo, Japan). After 3, 24, or 48 h of treatment with the different NP concentrations, cells were visualized, and phase contrast photographs of control and treated cells were obtained.

### 4.5. Cellular Viability

The MTT assay (according to Mossman, 1983, with some modifications) [80] was used to test the potential effects of ZnO NPs on the viability of glial A172 cells. Briefly, 2 × 10^4^ cells were seeded in 96-well plates and allowed to adhere for 24 h at 37 °C. Then, cells were treated with ZnO NPs or controls. After that, treatment suspensions were removed, and 100 μL of the MTT dye (500 μg/mL) dissolved in cell culture medium was added to each well and incubated at 37 °C for 4 h (protected from light). At the end of this period, the MTT solution was removed, 200 μL of DMSO was added to thoroughly solubilize purple formazan crystals, and the plate was kept away from light for an additional period of 10 min. Then, absorbance was measured at 570 nm using a SPECTROstar Nano microplate reader (BMG Labtech, Ortenberg, Germany), and values of the negative control (no treated cells) were used to correct the absorbance obtained in each tested condition. A minimum of three independent experiments, each one in triplicate, were carried out.

Before the experiments, the absorbance of ZnO NPs dispersed in water at 570 nm (wavelength used in the MTT assay) was checked. Since a progressive increase in the absorbance was observed from 10 µg/mL onwards (Supplementary Material, Figure S1), the potential interference of ZnO NPs with the standard protocol of the MTT assay was evaluated by a parallel set of experiments conducted without cells. As the results obtained demonstrated interactions of the ZnO NPs, not only with the dyes and reagents used in the MTT assay (light absorbance interference) but also with the detection method (catalytic interference) (Supplementary Material, Figure S2a), the protocol was modified to avoid this interference following the recommendations of Costa et al. [55]. Specifically, two additional washing steps with PBS were conducted after NP treatments, and centrifugation (1100× *g*, 10 min) was performed before absorbance reading, subsequently transferring the supernatants into a new 96-well plate. Interference testing was conducted again afterwards, confirming that NP interference was no longer present (Supplementary Material, Figure S2b). The modified MTT assay was then carried out to test ZnO NP effects on viability of A172 cells. The cytotoxicity parameter used was the percentage of cell viability, determined in each case from the absorbance data as follows: %Viability = (Abs sample)/(Abs negative control) × 100. Finally, the mean inhibitory concentration (IC_50_) was also calculated from the MTT assay results.

### 4.6. Zebrafish Husbandry and Embryo Collection

Adult zebrafish (*Danio rerio*, Fam. Cyprinidae) of the Tüpfel long fin (TL) line were kept in a standalone zebrafish housing rack under standard conditions that met FELASA guidelines (pH: 7.5 ± 1.0; conductivity: 700 ± 100 μS; temperature: 28.0 ± 1.0 °C; 14/10 h light/dark) [81]. Fish were fed on a mixture of commercial dry flakes twice a day and decapsulated live brine shrimp (*Artemia* sp.; JBL Artemio^®^ Pur) once a day. 

To obtain embryos, adult individuals were transferred to mating tanks at a 2:1 ratio (female/male). The next day, eggs were collected in Petri dishes containing sterile dechlorinated water with optimal parameters of 850 ± 50 µS conductivity and pH 7.3 ± 0.3. Petri dishes were maintained in an incubator at 28.5 ± 1 °C until use.

Zebrafish maintenance and experiments were performed following Spanish (RD 53/2013) and European (EU 2010/63) regulations on the protection of animals used for scientific purposes. The experiments involving animals were carried out complying with the replacement, reduction, and refinement (3Rs) principle in animal experimentation.

### 4.7. Fish Embryo Acute Toxicity Assay

Fertilized eggs at 2.5 hpf (256-cell stage) [29] were selected under a stereomicroscope and transferred to 4-well polystyrene plates (Nuclon™ Delta Surface; Thermo Fisher Scientific; 12 embryos in each well) containing 500 µL of the ZnO NP suspension per well. Seven concentrations of ZnO NPs were tested: 0.1, 0.5, 2.5, 10, 25, 50, and 100 µg/mL (i.e., 0.03–26.32 µg/cm^2^). Negative control embryos were exposed to fish water. The plates were kept in an incubator at 28.0 ± 1 °C with a light/dark cycle (14 h:10 h) for the duration of the experiments.

Four independent replicates, with 12 embryos per concentration, were carried out for each concentration in static experiments (no replacement of the suspensions). In addition, two replicates of semi-static experiments (daily medium replacement) were performed, as it was noticed that there was some sediment in the wells after 24 h. In all cases, the fertilization rate of the batch used in the experiments was ≥70%. Mortality in the negative controls was lower than 10% at 96 hpf. The sensitivity to 4.0 mg/L of 3.4-dichloroaniline of the zebrafish TL line used in the study was confirmed to be higher than 90% [79]. Based on the Embryo Acute Toxicity Test guidelines (FET) [79], five endpoints were recorded every 24 h up to 96 hpf: (a) coagulated fertilized eggs; (b) lack of heartbeat at 48 hpf; (c) presence of edema; (d) absence of somite formation; and (e) non detachment of the tailbud from the yolk sac. Although not an endpoint, the number of hatched embryos was also annotated every 24 h for the duration of the experiments. The percentage of survival and hatching was evaluated at the end of the 96 h period. Once the experiments were completed, all surviving embryos were euthanized by applying a humane method.

### 4.8. Morphological Analysis of Zebrafish Embryos

Morphological characteristics were examined at 96 hpf for embryos exposed to the highest concentration of ZnO NPs (100 µg/mL). The characteristics analyzed were: (1) body length (µm); (2) eye size (area normalized to body length); (3) morphology of the lower jaw (presence of protruding mouth); (4) pigmentation pattern (higher or lower pigmentation compared to controls). After euthanasia, embryos were transferred to 4% paraformaldehyde in 0.1 M phosphate buffer for at least 24 h. Embryos were then mounted in the lateral view in 1% low melting point agarose and images were taken using a 4X Plan Apo lens (0.2 NA; Nikon, Tokyo, Japan) of a bright field microscope (Eclipse 90i; Nikon) equipped with an Olympus DP71 color camera. Body length was considered as the distance from the mouth to the start of the caudal fin. The area of the eye was measured using Fiji [82], selecting a region of interest (ROI), and was normalized to body size (area to body length ratio).

### 4.9. Zinc Ions Released from the ZnO NPs

To quantify the Zn^2+^ ion concentrations released from the ZnO NPs. NP suspensions (0.1, 0.5, 2.5, 10, 25, 50, and 100 µg/mL) were incubated in complete cell culture medium for 3, 24, and 48 h at 37 °C in a humidified 5% CO_2_ environment, or in sterile dechlorinated tap water for 24, 48, 72, and 96 h at 28.5 ± 1 °C. Then, suspensions were centrifuged at 22,000× *g* for 30 min. The Zn^2+^ content in the supernatant was analyzed under standard operating conditions with FAAS (PerkinElmer Model 2380 atomic absorption/emission spectrometer, PerkinElmer Instruments equipped with a conventional nebulizer (glass impact bead) (PerkinElmer Inc., Waltham, MA, USA) and a Zn hollow cathode lamp (PerkinElmer) as the radiation source). During data acquisition, the spectrometer operating conditions were 213.9 nm resonance wavelength, 0.7 nm slit width and 12 mA intensity current. Samples were acidified with HNO_3_ to obtain a concentration of 1.0% (*w*/*v*) after dilution to 10.0 mL. 

Calibration (based on 1.0% (*w*/*v*) HNO_3_ aqueous standard solutions) and addition equations covering concentrations from 0 to 1.0 mg/L were assessed to check the matrix effect. The results showed that the slopes of the calibration (0.387 ± 0.045 L/mg) and standard addition (0.385 ± 0.028 L/mg) graphs are identical (*t*-test for a confidence level of 95.0%). Therefore, the matrix effect was not important, and, for Zn quantification, an aqueous calibration method could be applied. After performing different calibrations over five different days, good repeatability of the calibration slope was obtained. Linear dynamic range (R^2^ > 0.999) and limits of detection (LOD) (3 SDs criterion, where SD corresponds with standard deviation after analyzing eleven blanks) and limits of quantification (LOQs) (10 SDs criterion) were 5.6 and 13 µg/L, respectively. Inter-day precision and trueness of the method were assessed by analyzing two reference materials at different days: WS-PE-291099-03-01 and WS-PE-275150-15-01 Trace Metals Mix from AccuStandard (New Haven, CT, USA). Satisfactory results were obtained for inter-day precision (expressed as relative standard deviation, RSD%), being lower than 13%. Concerning trueness of the method, concentrations found in WS-PE-291099-03-01 (2910 ± 79 µg/L) and WS-PE-275150-15-01 (810 ± 32 µg/L) were in good agreement with the certified values (2649–3080 µg/L and 734–860 µg/L, for WS-PE-291099-03-01 and WS-PE-275150-15-01, respectively) after statistical evaluation by applying a *t*-test at a 95% confidence level for 8 degrees of freedom (calculated t values, t_cal_, achieved 1.63 and 1.15 for WS-PE-291099-03-01 and WS-PE-275150-15-01, respectively) and are lower than the tabulated t value (t_tab_) of 2.36. 

As a negative control, cell culture medium or sterile dechlorinated tap water without NPs but subjected to the same conditions was used. All experiments were performed in triplicate.

### 4.10. Toxicity of Zn^2+^ Ions 

In order to test whether the observed effects induced by ZnO NPs were due to Zn^2+^ ions released from ZnO NPs, A172 cells and zebrafish embryos were treated with ZnSO_4_ at concentrations set according to the results obtained in the FAAS experiments. They corresponded to 0.005, 0.01, 0.05, 0.1, 0.15, 0.2, 0.25, 0.3, and 0.5 mM for morphological analysis and MTT experiments in A172 cells, to 0.08 mM for morphological analysis of zebrafish embryos, and to 0.001, 0.005, 0.01, 0.02, 0.03, 0.04, 0.06, 0.08, and 0.1 mM for the FET assay.

### 4.11. Statistical Analyses

Statistical analyses were performed using SPSS for Windows statistical package (version 20.0). Distribution of the response variables departed significantly from normality (Kolmogorov–Smirnov goodness-of-fit test), and therefore nonparametric tests were considered adequate for the statistical analysis. Differences among groups were analyzed by Kruskal–Wallis test, with the Mann–Whitney *U*-test for two-by-two comparisons. The associations between two variables (linear concentration–response relationships) were analyzed by Pearson’s correlation. Experimental data were expressed as mean ± standard error, and a *p*-value of <0.05 was considered significant.

## 5. Conclusions

In this study, in vitro and in vivo effects of exposure to ZnO NPs were evaluated on human glial cells and zebrafish embryos, respectively. Even after removing the overestimation due to NP interference, ZnO NPs induced considerable cytotoxicity, including decrease in viability and cytoskeleton alterations, in glial cells in a dose- and time-dependent manner. However, exposure to these NPs did not induce morphological alterations or mortality in the exposed zebrafish embryos under any condition evaluated, and only a decrease in the percentage of hatching was observed at the highest dose tested. Free Zn^2+^ ions released from the ZnO NPs were not responsible for the decreased viability observed in glial cells, but they were likely to be responsible for the decreased hatching rate in zebrafish development. The results obtained in this work contribute to increasing the knowledge on the in vitro and in vivo toxicological potential of ZnO NPs.

## Figures and Tables

**Table 1:** Physicochemical characterization of ZnO NPs in A172 cell culture medium and dechlorinated water.

Cell culture medium	Time (h)	0	3	24	48	
	Hydrodynamic diameter (nm) ^a^	302.09 ± 0.84	315.01 ± 3.16	269.16 ± 1.36	273.97 ± 5.91	
	Zeta potential (mV) ^a^	−1.73 ± 4.05	2.79 ± 3.01	−20.25 ± 2.03	−13.46 ± 2.36	
Dechlorinated water	Time (h)	0	24	48	72	96
	Hydrodynamic diameter (nm) ^a^	301.36 ± 6.74	269.72 ± 0.12	242.46 ± 1.67	224.71 ± 2.38	238.15 ± 1.56
	Zeta potential (mV) ^a^	−23.23 ± 2.11	−18.22 ± 1.09	−26.73 ± 1.69	−22.22 ± 0.55	−22.49 ± 3.39

^a^ Mean ± standard deviation.

**Table 2:** IC_50_ values for treatment of A172 glial cells with ZnO NPs and ZnSO_4_, determined by MTT assay.

Exposure Time (h)	3	24	48
ZnO NPs IC_50_ (µg/mL) ^a^	120.51 ± 9.50	34.76 ± 1.92	17.08 ± 1.20
ZnSO_4_ IC_50_ (mM) ^a^	3.24 ± 1.43	0.41 ± 0.002	0.22 ± 0.001

^a^ Mean ± standard error.

## Data Availability

The data presented in this study are available from the corresponding author on request.

## Supplementary Materials

The following supporting information can be downloaded at: https://www.mdpi.com/article/10.3390/ijms241512297/s1.
