# Supplementary material for: Effects of Zinc Oxide Nanoparticle Exposure on Human Glial Cells and Zebrafish Embryos
Source: Int J Mol Sci. 2023 Aug 1;24(15):12297. doi: 10.3390/ijms241512297 (PMC10418813; doi:10.3390/ijms241512297)
Supplement: Supplementary file 1 [file ijms-24-12297-s001.zip › Figure S2 MTT interference.pdf]

Figure S2: Analysis of ZnO NP interference with MTT methodology following the traditional protocol, before (a) and after (b) implementing additional steps of washing and centrifugation in the original protocol (modified protocol): interference with dissolving agent (DMSO), interference with reaction product (formazan dissolved in DMSO), and catalytic interference. Bars represent mean standard error. *P<0.05, significant difference with regard to the control.

(a) Before correction **(b) After correction**

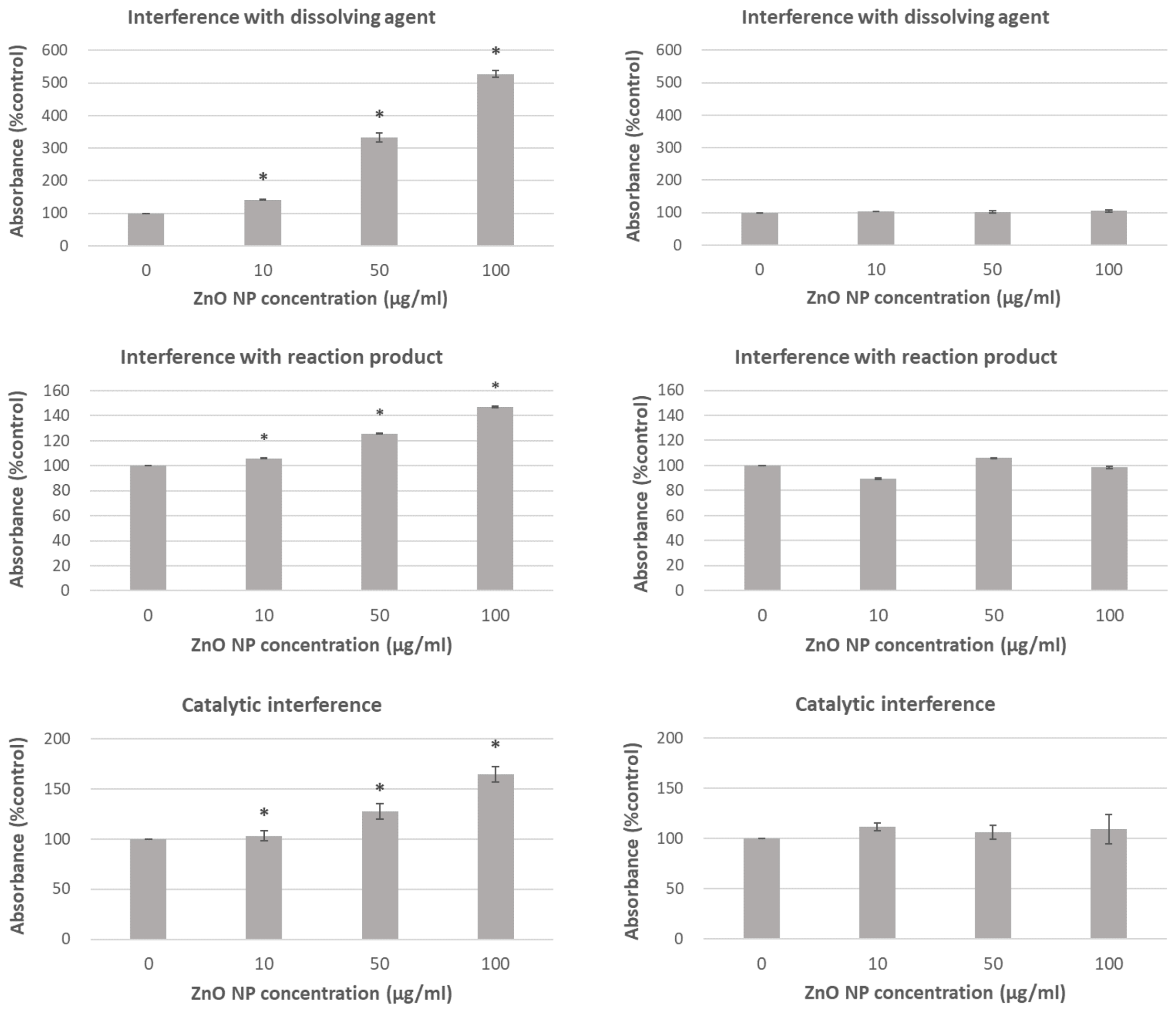

Possible interferences of ZnO NP with MTT assay experimental procedure were revealed with two sets of experiments in the absence of cells (1) to analyse the effect of NP presence on optical measurements (light-absorption interference) and (2) to assess possible NP reactivity with assay components (catalytic interference). Concentrations of ZnO NP selected for these experiments were 10, 50 and 100 µg/ml, and complete cell culture medium was used as negative control.

(1) Light-absorption interference was analysed in the presence of dissolving solution (DMSO) and reaction end-product (formazan dissolved in DMSO) as previously suggested by Kroll et al. [52]. End-product was obtained by incubating live A172 cells with MTT dye. The ZnO NP suspensions were prepared in DMSO and formazan dissolved in DMSO at the desired concentrations. Absorbance was measured at 570 nm using a SPECTROstar Nano (BMG Labtech) microplate reader.

(2) Catalytic interference: ZnO NP reactivity with MTT dye was determined by incubating 100 μl of NP suspensions prepared in cell culture medium with MTT reagent in the absence of cells for 4 h. At the end of the incubation, absorbance was measured at 570 nm.
