# Supplementary figures and images for: Effects of Zinc Oxide Nanoparticle Exposure on Human Glial Cells and Zebrafish Embryos
Source: Int J Mol Sci. 2023 Aug 1;24(15):12297. doi: 10.3390/ijms241512297 (PMC10418813; doi:10.3390/ijms241512297)

**Figure S1:** Absorbance at 570 nm of ZnO NP dispersed in water. Bars represent standard deviation.

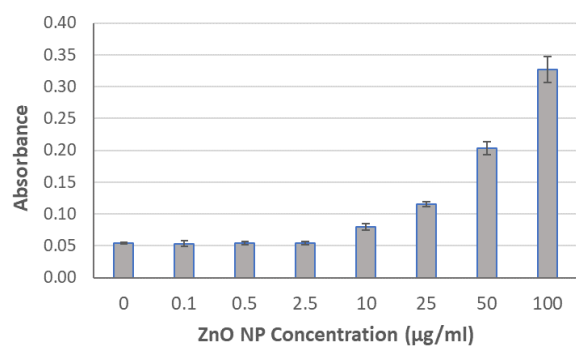

Supplement: Supplementary file 1 [file ijms-24-12297-s001.zip › Figure S1 absorbance in water.pdf]
